# FKBP51 overexpression in the corticolimbic system stabilizes circadian rhythms

**DOI:** 10.1016/j.cstres.2024.12.003

**Published:** 2024-12-12

**Authors:** Niat T. Gebru, David Beaulieu-Abdelahad, Danielle Gulick, Laura J. Blair

**Affiliations:** 1 Byrd Alzheimer's Center and Research Institute, Tampa, FL 33613, USA; 2 Department of Molecular Medicine, University of South Florida, Tampa, FL 33612, USA; 3 Research and Development, James A. Haley Veterans Hospital, Tampa, FL 33612, USA

**Keywords:** FKBP51, FKBP5, Circadian clock, Circadian rhythm sleep disorders, Transgenic mouse

## Abstract

Circadian rhythm disruptions have been associated with a wide range of health issues and complications, including an increased risk of circadian rhythm sleep disorders (CRSDs). CRSDs are common among individuals who have been through a traumatic event, particularly in those who have post-traumatic stress disorder (PTSD). Allelic variations in the gene encoding for FK506-binding protein 51 (FKBP51) can increase the susceptibility for PTSD and other stress-related disorders following trauma. At least one of these variants increases the levels of FKBP51 following stress through a glucocorticoid receptor-mediated process. Here, we used a mouse model that overexpresses human FKBP51 throughout the forebrain, rTgFKBP5, to investigate if elevated FKBP51 contributes to circadian rhythm disruption. Surprisingly, our findings indicate a greater rhythm amplitude and decreased rhythm fragmentation in rTgFKBP5 mice, particularly females, compared to controls. Female rTgFKBP5 mice also showed higher corticosterone levels basally and following stress exposure. Overall, this study associates FKBP51 overexpression with beneficial circadian rhythm outcomes.

## Highlights

• FKBP51 overexpression does not affect the core circadian mechanism.
• rTgFKBP5 mice exhibited strong rhythm amplitude and reduced intradaily variability.
• Female rTgFKBP5 mice have more pronounced corticosterone rhythms.

## Introduction

Circadian rhythms are highly conserved biological processes that regulate various physiological functions in living organisms, including humans.^1^ These rhythms play critical roles in regulating the sleep-wake cycle, hormone production, metabolism, and other vital functions^2^ and are generated by circadian clocks that are present in nearly all cells and tissues.^3^ Disruptions in circadian rhythms, such as those caused by shift work, jet lag, or exposure to artificial light at night, have been associated with a range of health issues,^4^ including an increased risk of circadian rhythm sleep disorders (CRSDs).^5^

CRSDs are an often-overlooked component of the sleep issues that are prevalent among individuals with post-traumatic stress disorder (PTSD), a psychiatric condition characterized by intrusive memories, hyperarousal, and avoidance behaviors following traumatic experiences.^4^, ^6^ Research in civilian cohorts has shown that over 70% of PTSD patients experience sleep disturbances, with this figure rising to over 90% among combat Veterans.^7^, ^8^ The relationship between PTSD and sleep disorders is bidirectional; sleep disruptions commonly occur in PTSD patients and correlate positively with the severity of the disorder, suggesting a causal role of PTSD in the development of sleep disorders.^9^, ^10^ In addition to factors that could entrain/affect the circadian clock, physical and psychological stress associated with mental disorders, such as PTSD, could also have a potential causal role in sleep disorders.^56^ Untreated sleep disorders can exacerbate PTSD symptoms, potentially diminishing treatment response and leading to poorer outcomes.^12^

Variants of the *FK506-binding protein 5* (*FKBP5*) gene that can potentially increase the levels of the FKBP51 protein have been implicated in numerous psychiatric disorders, including PTSD.^13^, ^14^ FKBP51, in part, is involved in regulating glucocorticoid receptor (GR)-mediated stress response *via* its interaction with heat shock protein 90 (Hsp90).^15^ FKBP51 binds to GR-Hsp90 complex and reduced GR’s affinity to steroid hormones, like CORT (cortisol in humans or corticosterone in mice).^16^, ^17^, ^18^ Upon stress, GR is activated by elevated levels of CORT. FKBP51 is replaced by other molecular chaperones, which mediates the nuclear translocation of GR.^16^ Nuclear GR regulates the transcription of glucocorticoid response element-containing genes, including *FKBP5*.^19^ Given that stress also influences circadian clock function and *Fkbp5* has been shown to be a mediator of circadian gene expression through GR,^20^ understanding the impact of elevated FKBP51 on circadian rhythms is critical.

In this study, we used a mouse model of FKBP51 overexpression to investigate the impact of elevated FKBP51 levels on circadian rhythms. Since circadian clock dysregulation can trigger sleep disturbances and critical clock genes are transcriptionally regulated by GR, we hypothesize that high levels of FKBP51 will disrupt normal circadian rhythms. To investigate this, circadian behavior was measured by monitoring wheel-running activity under different lighting conditions. CORT levels were also assessed at specific time points throughout the day to evaluate how FKBP51 overexpression in the corticolimbic system affects CORT dynamics, particularly in response to acute stress. Understanding the relationship between FKBP51 and circadian rhythms may offer insights into potential therapeutic strategies for managing stress-induced sleep disturbances and improving mental well-being.

## Results

### FKBP51 overexpression does not affect circadian period

**Fig. 1:**
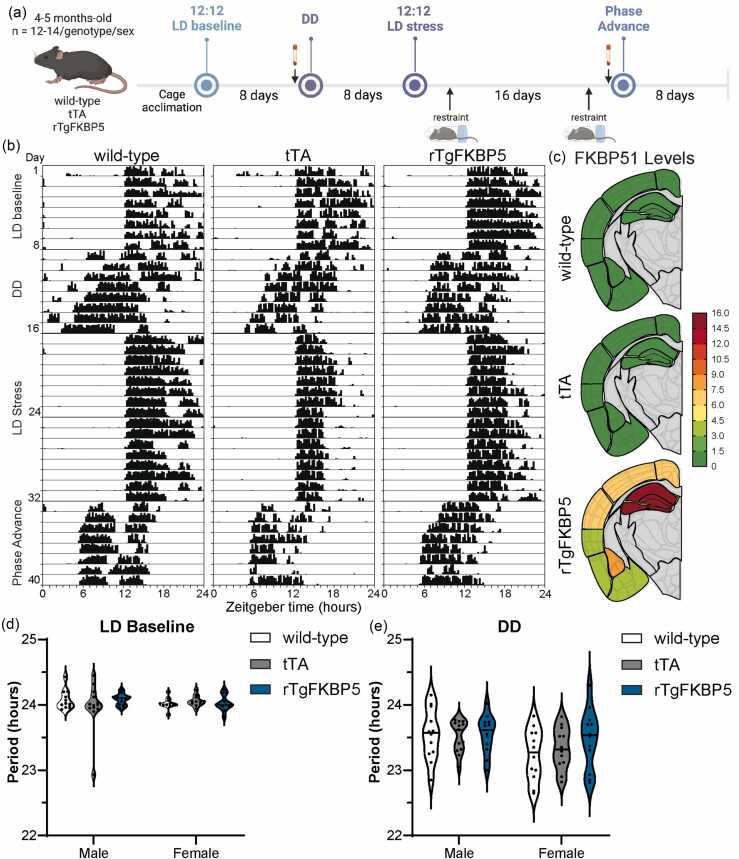
Circadian period is not affected by FKBP51 overexpression. (a) Male and female rTgFKBP5, aged 4–5 months, and control littermates (wild-type and tTA) were placed in circadian phenotyping cages. Wheel-running activity was measured as a proxy of circadian rhythmicity during different lighting conditions: LD baseline, DD, LD stress, and phase advance. Half of the mice were exposed to acute tube restraint stress at specific intervals to assess stress effects. Blood was collected from the submandibular vein at four-time points (ZT 0, ZT 6, ZT 12, and ZT 18) for corticosterone (CORT) assessment. Circadian period length during (b) representative actogram of daily wheel-running data displaying activity onset over a 24-h time period in wild-type, tTA, and rTgFKBP5 mice. (c) Semiquantitative heatmaps of the relative FKBP51 levels in major areas of the brain from wild-type, tTA, and rTgFKBP5 mice. Areas not quantified are shown in gray. Corresponding images and graphs are shown in Supplementary Figure 1. Circadian period length during (d) LD baseline and (e) DD in wild-type, tTA, and rTgFKBP5 male and female mice. Results represented as mean ± SEM (n = 8–12/sex/genotype). Data analyzed using SPSS MANOVA and ANOVA. Abbreviations used: DD, 24-h dark; FKBP51, FK506-binding protein 51; LD, 12-h light:12-hour dark; rTgFKBP5, transgenic mouse model overexpressing FKBP51; tTA, tetracycline transactivator; SEM, standard error of the mean; SPSS, Statistical Package for the Social Sciences; MANOVA, Multivariate analysis of variance; and ANOVA, Analysis of variance.

The main goal of this study was to characterize the circadian behavior profile of a mouse model that overexpresses *FKBP5* in the corticolimbic system (rTgFKBP5) to determine if elevated levels of FKBP51 in these brain regions promote an atypical rhythm compared to control littermates. Both wild-type and CamKIIα tetracycline transactivator (tTA) controls were used to differentiate the FKBP51-specific effects from any caused by the presence of the tTA. Circadian rhythms were assessed by recording the wheel-running activity of 4–5-month-old rTgFKBP5 (rTgFKBP5, n = 12 males and 12 females), wild-type (n = 13 males and 12 females), and tTA (n = 14 males and 12 females) mice in circadian phenotyping chambers under different lighting conditions (Figure 1(a)). The first activity measurement was recorded during standard 12-h light:12-h dark (LD baseline), followed by an assessment in 24-h darkness (DD) in the absence of external cues, also known as the free-running period (Figure 1(b)). Then, lighting conditions were returned to LD, during which two, 10-min tube restraints were conducted 5 days apart, 2 h after lights on. After 16 days in LD, activity was recorded in the presence of a 7-h phase advance (phase advance). Assessment of activity rhythms and circadian metrics, including period, amplitude, intradaily variability (IV), and interdaily stability (IS), was performed using ClockLab (Actimetrics), as detailed in methods. In line with previous reports using this model,^21^ FKBP51 levels were significantly higher in rTgFKBP5 mice compared to the controls used in this study (Figure 1(c) and Supplementary Figure 1).

**Fig. 2:**
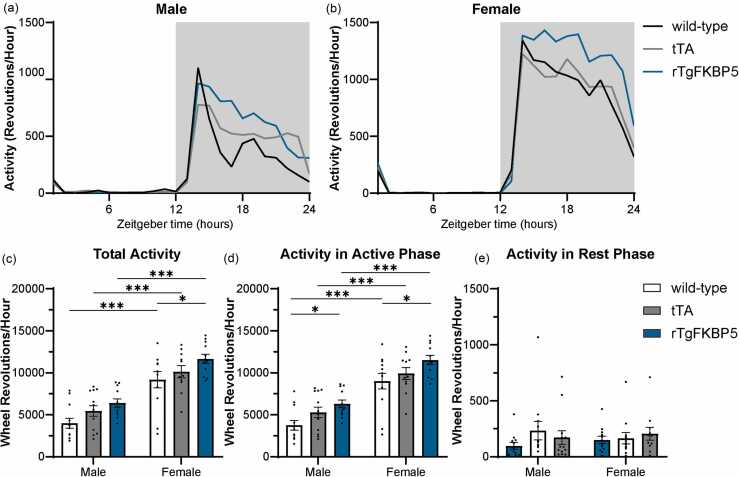
rTgFKBP5 mice have increased activity. Hourly activity profiles of wild-type, tTA, and rTgFKBP5 (a) male and (b) female mice generated in 60-min bins. Gray shading indicates when the lights are off. Wild-type, tTA, and rTgFKBP5 male and female mice were assessed for (c) total wheel-running activity counts during LD baseline as well as (d) activity during the active phase (lights off) and (e) activity during the rest phase (lights on). Results represented as mean ± SEM (n = 8–12/sex/genotype). Data analyzed using ANOVA with Tukey post hoc test. Statistical significance is indicated by *P < 0.05, ***P < 0.001. Abbreviations used: LD, 12-h light:12-hour dark; rTgFKBP5, transgenic mouse model overexpressing FKBP51; tTA, tetracycline transactivator; SEM, standard error of the mean; and ANOVA, Analysis of variance.

Male and female rTgFKBP5 mice showed no change in period length, the time it takes to complete one circadian cycle, compared to sex-matched tTA and wild-type controls (Genotype: F_2,65_ = 0.2069, *P* = 0.8136, Figure 1(d)). The period length during the free-running period (DD) was also unchanged by sex or genotype, although variability was higher overall in the absence of light cues (F_2,67_ = 0.7763, *P* = 0.4642, Figure 1(e)). As expected, since the free-running period is shorter (∼23.5 h) in mice,^22^ the free-running period in all groups during DD was shorter compared to LD and was unaffected by sex. The mean hourly activity in females was higher during the active phase compared to male mice (Figure 2(a) and (b)). The active phase occurs during Zeitgeber Time 12 (ZT12)-ZT24, with ZT0 as the start of circadian cues or “lights on” and ZT12 being “lights off” on a 12:12 LD schedule. Female total activity counts per hour were significantly higher compared to males (F_(1,67)_ = 73.180, *P* < 0.001, Figure 2(c)), which was due to increased activity during the active phase (alpha), as defined onset of activity from one day to the offset of the next (F_(1,66)_ = 86.9, *P* < 0.001, Figure 2(d)). No differences were observed during the rest phase (Rho), as defined by the offset of activity from one day to the onset of the next (Figure 2(e)). In addition, rTgFKBP5 mice showed higher activity compared to wild-type mice regardless of sex (F_(2,66)_ = 7.302, *P* < 0.001, Figure 2(d)).

### rTgFKBP5 mice display improved amplitude and intradaily variability

**Fig. 3:**
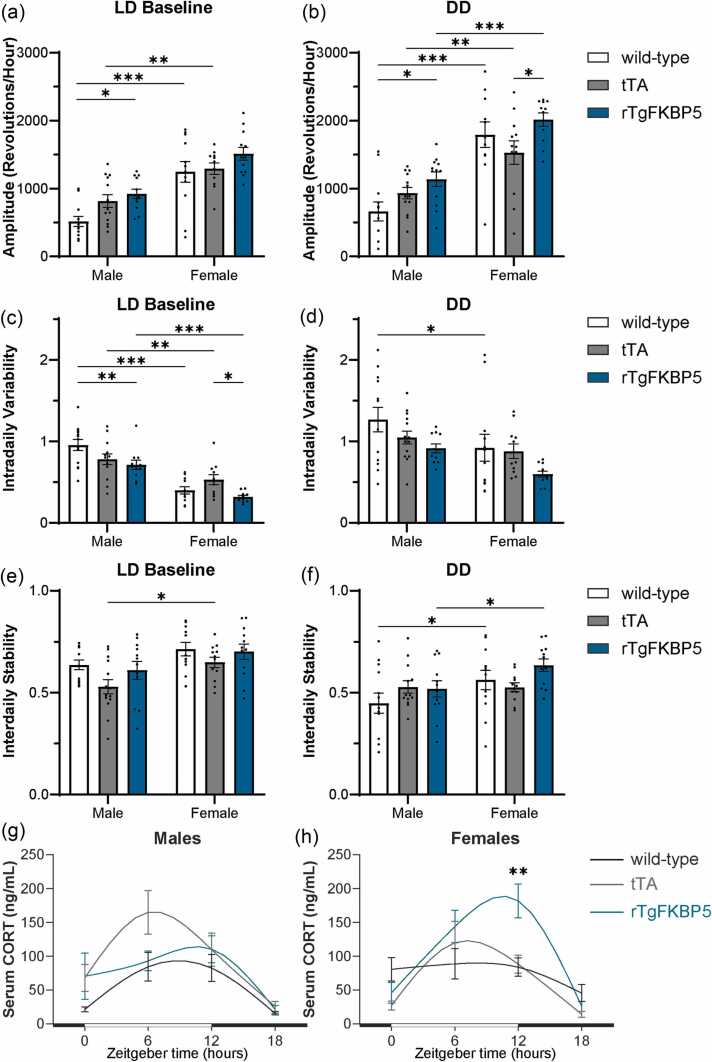
rTgFKBP5 mice show increased CORT, higher amplitude, and lower variability in females. Wheel-running activity was used to determine (a and b) rhythm amplitude, (c and d) intradaily variability, and (e and f) interdaily stability in male and female wild-type, tTA, and rTgFKBP5 mice during entrainment (LD baseline) and the free-running period (DD), as indicated. Data analyzed using SPSS MANOVA and ANOVA with Tukey post hoc test. Results represented as mean ± SEM (n = 10–12/sex/genotype). Serum CORT levels from blood collections at four-time points (ZT 0, ZT 6, ZT 12, and ZT 18) in (g) male and (h) female wild-type, tTA, or rTgFKBP5 mice. Open and filled bars represent 12 h light and dark cycles. Results represented as mean ± SEM (n =6–7/sex/genotype/ZT). Time is denoted as Zeitgeber Time (ZT), where ZT 0 = lights on and ZT 12 = lights off. Statistical significance is indicated by *P < 0.05, **P < 0.01, ***P < 0.001. Abbreviations used: DD, 24-h dark; LD, 12-h light:12-hour dark; rTgFKBP5, transgenic mouse model overexpressing FKBP51; tTA, tetracycline transactivator; SEM, standard error of the mean; SPSS, Statistical Package for the Social Sciences; MANOVA, Multivariate analysis of variance; and ANOVA, Analysis of variance.

During LD baseline and DD, rTgFKBP5 male mice showed significantly increased rhythm magnitude, which is quantified by rhythm amplitude or the difference between the highest activity to the lowest activity, compared to wild-type (LD baseline: *P* < 0.05, DD: *P* < 0.05) but not tTA males (Figure 3(a) and (b)), which suggests the tTA transgene contributes to this effect. In rTgFKBP5 females, the amplitude was significantly increased compared to tTA females during DD (*P* < 0.05, Figure 3(a) and (b)). Interestingly, significant sex effects on amplitude were present consistently, which were increased in females compared to males in both LD (F_1,66_ = 55.10, *P* < 0.0001) and DD (F_1,65_ = 61.85, *P* < 0.0001), regardless of genotype. Together, these data suggest there is a stronger circadian rhythm amplitude in female mice, which was strengthened by the overexpression of FKBP51 in the absence of light cues.

Rhythm fragmentation was measured by IV. Female mice showed a significant decrease in IV during LD in females regardless of genotype compared to their respective male counterparts (F_(1,64)_ = 71.58, *P* < 0.001, Figure 3(c)). A significant decrease in IV was measured in male rTgFKBP5 mice, but only compared to male wild-type mice (*P* < 0.0001). On the contrary, IV was decreased in female rTgFKBP5 mice compared to female tTA mice (*P* < 0.05), possibly suggesting sex-specific influence of the tTA transgene. IV was significantly decreased in all rTgFKBP5 genotype compared to controls during DD (F_(2,69)_ = 3.393, *P* = 0.039, Figure 3(d)). IS was unchanged among the genotypes during LD baseline. During DD, however, it was significantly increased females compared to males in both wild-type and rTgFKBP5 mice in DD (*P* < 0.05, Figure 3(e) and (f)).

### FKBP51 overexpression in female mice increases CORT dynamics

Since CORT levels impact rhythmic gene expression and are closely linked to the circadian clock,^23^, ^24^ serum CORT levels were measured on the last two days of LD baseline at four-time points to evaluate the dynamic changes throughout the day – 6 am (ZT0, lights-on, start of the rest phase), 6 pm (ZT12, lights-off, start of the active phase) and as well as 12 pm (ZT06), and 12 am (ZT18). To achieve this, blood collection was carried out at two-time points from each mouse over a 48-h period, either at ZT0 and ZT12 or ZT06 and ZT18). As expected, significant rhythmicity in CORT levels was observed in male (F_(3,63)_ = 13.96, *P* < 0.001) and female (F_(3,58)_ = 18.71, *P* < 0.001) mice throughout the circadian day. Male rTgFKBP5 and wild-type mice displayed similar CORT rhythms, peaking shortly before ZT 12 (Figure 3(g)). However, this was less pronounced in the wild-type females, who showed steady levels throughout the day, which troughed at ZT 18 (Figure 3(h)). rTgFKBP5 females, however, had a more dynamic rhythm with peak levels at ZT 12, which were significantly higher compared to wild-type and tTA females (*P* < 0.01). Of note, the peak CORT in tTA mice was shifted earlier to ZT 6 regardless of sex (Figure 3(g) and (h)).

### Acute stress increases CORT sensitivity of rTgFKBP5 female mice but does not affect circadian rhythm profile

**Fig. 4:**
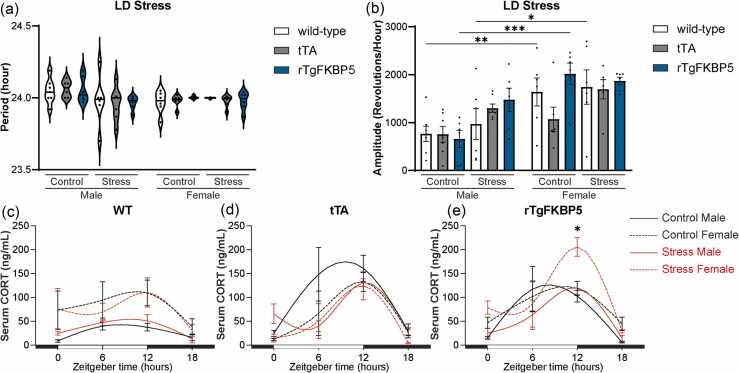
Acute stress enhances CORT sensitivity in rTgFKBP5 female mice but does not alter the circadian rhythm profile. Wheel-running activity was used to determine (a) Period and (b) amplitude in control and stress-exposed male and female wild-type, tTA, and rTgFKBP5 mice during LD stress. Data analyzed using SPSS MANOVA and ANOVA with Tukey post hoc test. Results represented as mean ± SEM (n = 5–7/sex/genotype/stress). Serum corticosterone (CORT) levels from blood collections at four-time points (ZT 0, ZT 6, ZT 12, and ZT 18) in (c) wild-type, (d) tTA, and (e) rTgFKBP5 males and females. Open and filled bars represent 12 h light and dark cycles. Results represented as mean ± SEM (n = 5–7/sex/genotype/stress/ZT). Time is denoted as zeitgeber time (ZT), where ZT 0 = lights on and ZT 12 = lights off. Statistical significance is indicated by *P < 0.05, **P < 0.01, ***P < 0.001. Abbreviations used: LD, 12-h light:12-hour dark; rTgFKBP5, transgenic mouse model overexpressing FKBP51; tTA, tetracycline transactivator; SEM, standard error of the mean; SPSS, Statistical Package for the Social Sciences; MANOVA, Multivariate analysis of variance; and ANOVA, Analysis of variance.

To understand the effects of stress on *FKBP5* overexpression and its subsequent impact on circadian regulation (LD stress), half of the mice in each group (rTgFKBP5, n = 6 males and 6 females, wild-type, n = 7 males and 6 females, and tTA n = 6 males and 6 females) were exposed to a 10-min tube restraint test, and then, after 4 days, blood was taken to assess the effects of acute stress on CORT. Stress did not affect circadian period (Figure 4(a)), but sex-specific effects were observed in the rhythm amplitude (F_(1,63)_ = 27.88, *P* < 0.001). Significant differences were measured between male and female rTgFKBP5 nonstress groups (*P* < 0.001) that is not present after stress exposure (Figure 4(b)). IV and IS were unchanged by stress regardless of genotype (Supplementary Figure 2(a) and (b)).

The male wild-type groups had relatively stable CORT levels with a slight peak between ZT 6 and ZT 12 (Figure 4(c)). On the contrary, wild-type females showed CORT fluctuations with peaks at ZT 12 for both stressed and control groups. Stress increased CORT levels slightly but not significantly in wild-type mice. Stress effects were not evident in the tTA groups, which showed the lowest CORT levels during the early hours and peaking at ZT 12 regardless of sex (Figure 4(d)). CORT peaked in rTgFKBP5 control males between ZT 6 and ZT 12, which was shifted to ZT 12 in the stress-exposed group. In female rTgFKBP5 mice, stress significantly increased CORT levels compared to controls, which was especially noticeable at ZT 12 (*P* ≤ 0.05, Figure 4(e)). Overall, this suggests that female rTgFKBP5 mice may have enhanced sensitivity to stress.

**Fig. 5:**
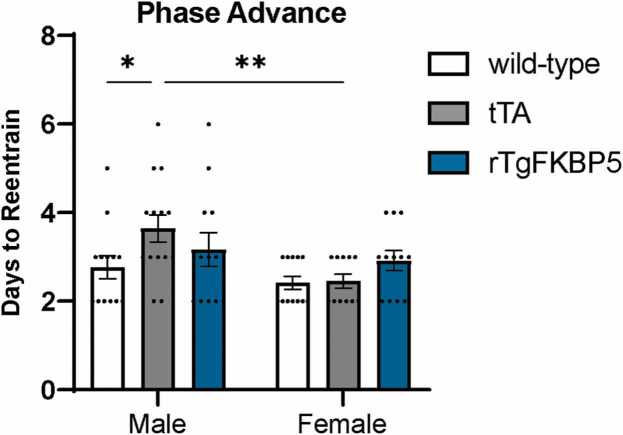
FKBP51 overexpression does not alter resynchronization to phase shifts. rTgFKBP5, tTA, and wild-type mice were subjected to a 7-h phase advance and the time needed (days) to resynchronize to the new light-dark cycle was measured. Results represented as mean days ± SEM (n = 10–13/sex/genotype). Data analyzed using ANOVA with Tukey post hoc test. Statistical significance is indicated by *P < 0.05, **P < 0.01. Abbreviations used: FKBP51, FK506-binding protein 51; rTgFKBP5, transgenic mouse model overexpressing FKBP51; tTA, tetracycline transactivator; SEM, standard error of the mean; and ANOVA, Analysis of variance.

Next, the ability of the circadian period to entrain to phase shifts was tested by introducing a phase advance in which the lights turn on and off 7 h earlier in the day. Analysis was preformed to determine how many days each mouse took to adjust to the new onset time. While effects of FKB51 were not observed in either sex, tTA male mice took longer to adjust to the new light onset compared to wild-type male mice (*P* ≤ 0.05) and tTA female mice (*P* ≤ 0.01) (Figure 5).

## Discussion

This study aimed to evaluate the effects of elevated FKBP51 levels in the corticolimbic system on circadian behavior and CORT rhythm. Our findings reveal that FKBP51 overexpression can significantly impact circadian rhythms, particularly in relation to sex differences and stress response. Actigraphy-estimated rest and activity behaviors were notably changed in the rTgFKBP5 mice compared to controls. Specifically, rTgFKBP5 mice exhibit a more robust circadian rhythm indicated by increased amplitude and lower rhythm fragmentation, contrary to our initial expectations. Altered diurnal CORT rhythms were also observed in rTgFKBP5 mice, which were further affected in females following acute stress exposure. Overall, this suggests that FKBP51 can influence circadian and CORT rhythms in a sex-dependent and stress-dependent manner.

While many of the outcomes remained unchanged by elevated FKBP51, rTgFKBP5 male and female mice showed higher rhythm amplitude and lower IV in males. The enhanced amplitude, especially during the free-running period, suggests that elevated FKBP51 levels may enhance circadian rhythm dynamics through its regulatory role on glucocorticoid signaling pathways. Higher rhythm amplitudes are often associated with lower IV, which indicates a more stable and consolidated rest-activity pattern with fewer transitions between rest and activity periods.^25^ These findings were unexpected, since increased FKBP51 has been associated with several adverse outcomes,^21^, ^26^ and lower IV is thought to be beneficial.^11^ However, our previous research showed that rTgFKBP5 mice were protected from depressive-like symptoms induced by early life stress.^27^ Although *FKBP5* single nucleotide polymorphisms are linked to increased susceptibility to stress-related disorders, like depression in humans,^28^, ^29^ they also increase responsiveness to antidepressants,^30^ likely due to altered regulation of autophagy pathways.^31^, ^32^ Interestingly, there is growing evidence to support a regulatory and reciprocal role of circadian rhythms on the function of autophagy.^33^, ^34^

A study by Hausl and colleagues^35^ explored the association of FKBP51 in the mediobasal hypothalamic area with autophagy and metabolism. Interestingly, this study demonstrated FKBP51 overexpression protects against diet-induced obesity, while cell-type specific deletion of *Fkbp5* in the hypothalamus was associated with increased body weight and obesity.^35^, ^36^ However, whole-body *Fkbp5* deletions have been generally associated with improved metabolic outcomes, illustrating the tissue-specific roles of FKBP51. Hausl and colleagues^35^ suggested that FKBP51 overexpression levels (dose) are critical and contribute to these conflicting observations in metabolic phenotypes. It is also worth noting that, in a recent study by our group, circadian rhythms and associated core clock proteins were unaffected by *Fkbp5* ablation,^37^ suggesting the increased stability in circadian rhythms observed with FKBP51 overexpression are not reversed by its absence. Our findings add to the literature showing that FKBP51 plays a multifaceted role in the regulation of many pathways,^38^ highlighting the need for more studies to elucidate the relationship between FKBP51 levels and functional implications.

This study represents the first examination of CORT levels at four distinct time points in the rTgFKBP5 model, in order to achieve a more comprehensive picture of CORT diurnal dynamics during sustained FKBP51 levels. Female rTgFKBP5 mice exhibited elevated CORT levels throughout most of the subjective night. Interestingly, in *FKBP5*-humanized mice the females also demonstrate altered CORT rhythms.^39^ However, instead of having boosted CORT levels during times of activity, female mice with the *FKBP5* risk variant showed higher levels in the morning. Taken together, this suggests that females are more sensitive to altered CORT dynamics through FKBP51. In humans, stressors, such as sleep deprivation, can lead to elevated nighttime CORT levels at.^40^ Chronic sleep disruption, another type of stressor, has been shown to alter CORT secretion patterns,^41^ specifically leading to elevated nighttime CORT levels.^42^ The relationship between CORT and sleep disruptions is bidirectional, with elevated CORT secretion correlating with reduced sleep quality,^43^ and poor sleep quality associating with disrupted CORT rhythm.^44^ While this study focused on the impact of acute stress, further research is necessary to investigate the effects of FKBP51 on CORT feedback regulation in the context of chronic stress. On a similar note, although circadian rhythms are not affected by acute stress in this model, the effects of long-term stress may differ, as chronic stress is known to disrupt the circadian clock.^45^

Interestingly, the circadian period remained consistent in rTgFKBP5 mice compared to littermate controls. The lack of significant differences in period length, despite FKBP51 overexpression, suggests that while FKBP51 may influence other aspects of circadian behavior, it does not disrupt the fundamental timing mechanisms of the circadian clock. However, it cannot be excluded that this may be due to the lack of dynamic FKBP51 expression in this model, since the *FKBP5* transgene is regulated by the CamKIIα promoter.^21^ This regulation may limit the effects on circadian biology and can promote overexpression in brain regions that typically express lower levels of FKBP51. Therefore, the exogenous *FKBP5* transgene may not regulate the stress response feedback loop in the same manner as endogenous FKBP51, which has been shown to exhibit circadian expression across multiple datasets and tissues.^20^ It is also important to note that the neurons in the suprachiasmatic nucleus, home of the master clock, do not express GR,^46^ however, there are still indirect feedback mechanisms linking CORT and circadian rhythms.^47^ This biological separation may act as a buffer to preserve circadian rhythm function, even during periods of altered CORT rhythms or heightened organismal stress. However, FKBP51 has been shown to be involved in multiple feedback loops,^38^ suggesting that its influence extends beyond direct regulation of the GR pathway.

Sex differences in activity levels were also observed, with female mice exhibiting higher overall activity compared to males during the active phase. Similar to our rhythm amplitude outcomes, other studies have reported stronger circadian rhythms in female mice compared to males, particularly during the active phase.^45^ These differences are thought to be influenced by underlying biological processes, such as hormonal variations, particularly those related to the estrous cycle, as well as differences in metabolism and energy expenditure between male and female mice.^48^, ^49^

An important consideration in this study is the use of tTA mice as controls, in addition to the wild-type groups. This was done to account for potential behavioral or molecular effects induced by the CamKIIα promoter, since there are known connections between CamKIIα and circadian rhythm pathways. More specifically, CaMKIIα signaling plays a role in activating E-box-dependent gene expression, which is essential for maintaining circadian rhythms.^50^ Furthermore, CaMKII directly phosphorylates CLOCK, a key regulator of circadian gene expression, thereby affecting the oscillations of the circadian clock.^51^ Because both control groups were included in this study, FKBP51-specific effects can be differentiated from those specific to the tTA transgene.

## Conclusion

In summary, the results of this study contribute to a growing body of literature that examines the interaction among FKBP51, circadian rhythms, and stress response. The consistent circadian period across genotypes, coupled with enhanced amplitude and sex-specific differences in activity and CORT dynamics, highlights the multifaceted role of FKBP51 in circadian biology and its potential implications for understanding stress-related disorders. Understanding this connection paves the way for developing therapies to mitigate stress-induced circadian disruptions, especially for PTSD and other stress-related disorders.

## Materials and methods

### Animals

All studies were conducted following the guidelines set by the University of South Florida’s Institutional Animal Care and Use Committee in accordance with the Association for Assessment and Accreditation of Laboratory Animal Care international regulations. The rTgFKBP5 mice were generated by crossing FVB mice containing a single copy of the human FKBP5 gene with 129S6 CamKIIα-tTA mice. The offspring, rTgFKBP5, constitutively express high FKBP51 throughout the forebrain under the CAMKIIα promoter (Tet-OFF).^21^ rTgFKBP5, as well as wild-type and tTA control littermates (n = 12–14/sex/genotype), were bred in-house. Both tTA and wild-type littermates were used, so the effects of the CamKIIα-tTA can be differentiated from FKBP51-dependent effects since some differences have been reported in this line previously.^52^ They were group-housed and aged to 4 months with a 12-h light/dark cycle and ad libitum access to food and water until the start of this study.

### Assessment of circadian activity and data acquisition

4–5-month-old mice were individually housed in circadian phenotyping chambers (Tecniplast, West Chester, PA, USA). Wheel-running activity was measured to assess circadian rhythmicity during 12-h light/dark (LD baseline), followed by 24-h darkness (DD), and then another cycle of LD for re-entrainment before exposing them to stress (LD stress), and finally a 7-h phase shift period (Phase Advance). Each experimental light period lasted 7–9 days to give the mice ample time to entrain to the new settings. On day 5 of the LD stress cycle, half of the mice were subjected to a 10-min acute tube restraint at ZT 2, and controls were placed in empty cages. This was carried out by first determining the actual onset of activity and then administering restraint within a 1-h window of ZT 2. Welfare checks during the dark cycle utilized infrared goggles and a red light (630 nm, wavelength below the threshold for mouse perception). Data were recorded in 5-min bins using Scurry Activity Monitor (Lafayette Instruments, Lafayette, IN, USA) and activity graphs (actograms) generated using ClockLab software (Actimetrics, Wilmette, IL, USA). The circadian period for each mouse was computed from ClockLab by calculating the slope of a regression line based on successive circadian activity onsets using the Chi-squared periodogram (Bushell and Sokolove method). IS and IV were computed using nonparametric calculations based on the ratio of the average square error of hourly means from the grand mean divided by the sample variance. IS measures how well the daily activity patterns resemble one another from one day to the next and ranges from 0, representing inconsistent rhythm, to 1, representing high rhythm consistency. IV measures rhythm fragmentation or frequency of transitions between rest and activity within 24 h and ranges from 0, corresponding to stable rhythms, to 2, representing greater rhythm fluctuations. These are computed by the nonparametric circadian rhythm analysis module within ClockLab. Phase advance was calculated as the number of days each mouse took to adjust their activity onset 7 h earlier.

### Measurement of CORT dynamics

Blood was collected from the submandibular vein at the end of the LD and LD Stress periods at four-time points (ZT 0, ZT 6, ZT 12, and ZT 18), which was determined based on the onset of activity obtained from ClockLab the day before blood collection. The cohorts were divided into two for blood collections. Two collections, at either ZT0 and ZT12 or ZT 6 and ZT18, were carried out for each mouse over the course of 48 h. All collections were done within 30 min of the assigned ZT. Serum was separated from whole blood using serum separator tubes (BD, Franklin Lakes, NJ, USA) by centrifugation for 15 min at 2000g at 4ºC. Serum CORT was diluted 1:40 and quantified using an ELISA kit (Cat# ADI-901-097, Enzo Life Sciences, Farmingdale, NY, USA), as recommended by the manufacturer.

### Immunohistochemical staining and analysis

After circadian assessment was complete, mice were overdosed with Euthasol and transcardially perfused with saline. Coronal sections were generated using a sliding microtome from paraformaldehyde-fixed hemibrains following sucrose cryoprotection. FKBP51 Immunohistochemical staining was performed as described^21^ using FKBP51 (1:300, Cat# AF4094, R&D Systems, Minneapolis MN, USA) primary antibody with donkey anti-goat IgG biotin secondary antibody (1:3000, Cat# 1031-08, SouthernBiotech, Birmingham AL, USA). Imaging was performed using a Zeiss Axio Scan.Z1 slide scanner (ZEISS Microscopy, Munich, Germany). Brightfield images were then opened in QuPath Bioimage Analysis software v0.5.1.^53^ The hippocampus, cortex (somatosensory and auditory areas), entorhinal cortex (including the ectorhinal area), and amygdala were manually annotated, and relative optical densities quantified using the same trained pixel classifier. Microsoft Excel following a previously described macro^54^ with images adapted from the Allen Mouse Brain Atlas.^55^

### Statistical analysis

All circadian measurement variables (period, amplitude, IV, and IS) and activity counts were computed based on activity onsets in 60 min bins using ClockLab analysis software v6.1. Resynchronization to a new LD cycle, after 7-h phase advance, was considered complete when the new activity onset took place >6.5 h earlier. The number of days required to resynchronize was calculated for each animal. Group outliers were identified by Grubb’s test. Statistical Package for the Social Sciences v29 (Chicago, IL, USA) was used to analyze behavior data generated in ClockLab software using multivariate analysis of variance and analysis of variance (ANOVA). Tukey’s post hoc test was used to identify significant differences based on *P* < 0.05. GraphPad Prism 10.3.1 (GraphPad Software, San Diego, CA, USA) was used to analyze Phase advance (two-way ANOVA) and immunohistochemical (one-way ANOVA) data with Tukey’s post hoc test as well as generate graphs.

## Author contributions

**Laura Blair:** Writing – review & editing, Writing – original draft, Visualization, Supervision, Project administration, Methodology, Funding acquisition, Formal analysis, Data curation, Conceptualization. **Danielle Gulick:** Writing – review & editing, Supervision, Methodology, Formal analysis, Conceptualization. **David Beaulieu-Abdelahad:** Investigation. **Niat T. Gebru:** Writing – review & editing, Writing – original draft, Visualization, Methodology, Investigation, Formal analysis, Data curation.

## Declaration of Generative AI and AI-assisted technologies in the writing process

During the preparation of this work the author(s) used Microsoft Copilot and Grammarly to improve the readability of the manuscript as well as scite.ai to identify additional relevant references. After using these tools, the authors reviewed and edited the content as needed and take full responsibility for the content of the publication.

## Declarations of interest

The authors declare the following financial interests/personal relationships, which may be considered as potential competing interests: Laura Blair reports financial support was provided by the National Institutes of Health, the Alzheimer’s Association, and the US Department of Veterans Affairs. Laura Blair has patent #US20150327523 A1 issued to Laura Blair. Laura Blair is a Senior Editor at Cell Stress and Chaperones. The other authors declare that they have no known competing financial interests or personal relationships that could have appeared to influence the work reported in this paper.

## Data Availability

Data will be made available on request.

## Footnotes

Appendix A Supplementary data associated with this article can be found in the online version at doi:10.1016/j.cstres.2024.12.003.

## References

[1] Patke A., Young M.W., Axelrod S. (2020). Molecular mechanisms and physiological importance of circadian rhythms. Nat Rev Mol Cell Biol.

[2] Finger A.M., Dibner C., Kramer A. (2020). Coupled network of the circadian clocks: a driving force of rhythmic physiology. FEBS Lett.

[3] Mezan S., Feuz Jean D., Deplancke B., Kadener S. (2016). PDF Signaling Is an Integral part of the Drosophila Circadian Molecular Oscillator. Cell Rep..

[4] Brown L.A., Fisk A.S., Pothecary C.A., Peirson S.N. (2019). Telling the time with a broken clock: quantifying circadian disruption in animal models. Biology.

[5] Zhu L., Zee P.C. (2012). Circadian rhythm sleep disorders. Neurol Clin.

[6] Hou Y., Liu L., Chen X., Li Q., Li J. (2020). Association between circadian disruption and diseases: a narrative review. Life Sci.

[7] Ohayon M.M., Shapiro C.M. (2000). Sleep disturbances and psychiatric disorders associated with posttraumatic stress disorder in the general population. Compr Psychiatry.

[8] Seelig A.D., Jacobson I.G., Smith B. (2010). Sleep patterns before, during, and after deployment to Iraq and Afghanistan. Sleep.

[9] Agorastos A., Olff M. (2020). Traumatic stress and the circadian system: neurobiology, timing and treatment of posttraumatic chronodisruption. Eur J Psychotraumatol.

[10] Myers B.L., Badia P. (1995). Changes in circadian rhythms and sleep quality with aging: mechanisms and interventions. Neurosci Biobehav Rev.

[11] Gonçalves B.S.B., Adamowicz T., Louzada F.M., Moreno C.R., Araujo J.F. (2015). A fresh look at the use of nonparametric analysis in actimetry. Sleep Med Rev.

[12] Sandahl H., Jennum P., Baandrup L. (2017). Treatment of sleep disturbances in trauma-affected refugees: study protocol for a randomised controlled trial. Trials.

[13] Binder E., Salyakina D., Lichtner P. (2004). Polymorphisms in *FKBP5* are associated with increased recurrence of depressive episodes and rapid response to antidepressant treatment. Nat Genet.

[14] Willour V., Chen H., Toolan J. (2009). Family-based association of *FKBP5* in bipolar disorder. Mol Psychiatry.

[15] Pratt W.B. (1993). The role of heat shock proteins in regulating the function, folding, and trafficking of the glucocorticoid receptor. J Biol Chem.

[16] Wochnik G.M., Rüegg J., Abel G.A., Schmidt U., Holsboer F., Rein T. (2005). FK506-binding proteins 51 and 52 differentially regulate dynein interaction and nuclear translocation of the glucocorticoid receptor in mammalian cells. J Biol Chem.

[17] Denny W.B., Valentine D.L., Reynolds P.D., Smith D.F., Scammell J.G. (2000). Squirrel monkey immunophilin FKBP51 is a potent inhibitor of glucocorticoid receptor binding. Endocrinology.

[18] Sabbagh J.J., Cordova R.A., Zheng D. (2018). Targeting the FKBP51/GR/Hsp90 complex to identify functionally relevant treatments for depression and PTSD. ACS Chem Biol.

[19] Muzikar K.A., Nickols N.G., Dervan P.B. (2009). Repression of DNA-binding dependent glucocorticoid receptor-mediated gene expression. Proc Natl Acad Sci USA.

[20] Yan J., Wang H., Liu Y., Shao C. (2008). Analysis of gene regulatory networks in the mammalian circadian rhythm. PLoS Comput Biol.

[21] Blair L.J., Criado-Marrero M., Zheng D. (2019). The disease-associated chaperone FKBP51 impairs cognitive function by accelerating AMPA receptor recycling. Eneuro.

[22] Eckel-Mahan K., Sassone-Corsi P. (2015). Phenotyping circadian rhythms in mice. Curr Protoc Mouse Biol.

[23] Bering T., Hertz H., Rath M.F. (2020). Rhythmic release of corticosterone induces circadian clock gene expression in the cerebellum. Neuroendocrinology.

[24] Roa S.L.R., Martinez E.Z., Martins C.S., Antonini S.R., de Castro M., Moreira A.C. (2017). Postnatal ontogeny of the circadian expression of the adrenal clock genes and corticosterone rhythm in male rats. Endocrinology.

[25] Li P., Gao L., Gaba A. (2020). Circadian disturbances in Alzheimer’s disease progression: a prospective observational cohort study of community-based older adults. The Lancet Healthy Longevity.

[26] Gebru N.T., Hill S.E., Blair L.J. (2024). Genetically engineered mouse models of FK506-binding protein 5. J Cell Biochem.

[27] Criado-Marrero M., Smith T.M., Gould L.A. (2020). FKBP5 and early life stress affect the hippocampus by an age-dependent mechanism. Brain Behav Immu Health.

[28] Cattaneo A., Gennarelli M., Uher R. (2013). Candidate genes expression profile associated with antidepressants response in the GENDEP study: differentiating between baseline ‘predictors’ and longitudinal ‘targets’. Neuropsychopharmacology.

[29] Lekman M., Laje G., Charney D. (2008). The FKBP5-Gene in depression and treatment response—an association study in the sequenced treatment alternatives to relieve depression (STAR*D) Cohort. Biol Psychiatry.

[30] Zhang Y., Yue W., Li J. (2024). The association of FKBP5 gene polymorphism with genetic susceptibility to depression and response to antidepressant treatment - a systematic review. BMC Psychiatry.

[31] Gassen N.C., Hartmann J., Zschocke J. (2014). Association of FKBP51 with priming of autophagy pathways and mediation of antidepressant treatment response: evidence in cells, mice, and humans. PLoS Med.

[32] Silva S., Bicker J., Falcão J., Fortuna A. (2021). Antidepressants and circadian rhythm: exploring their bidirectional interaction for the treatment of depression. Pharmaceutics.

[33] Juste Y.R., Kaushik S., Bourdenx M. (2021). Reciprocal regulation of chaperone-mediated autophagy and the circadian clock. Nat Cell Biol.

[34] Di T., Zhou Z., Liu F., Chen Y., Wang L. (2024). Autophagy and circadian rhythms: interactions and clinical implications. Biocell.

[35] Häusl A.S., Bajaj T., Brix L.M. (2022). Mediobasal hypothalamic FKBP51 acts as a molecular switch linking autophagy to whole-body metabolism. Sci Adv.

[36] Brix L.M., Toksöz I., Aman L. (2022). Contribution of the co-chaperone FKBP51 in the ventromedial hypothalamus to metabolic homeostasis in male and female mice. Molecular Metabolism.

[37] Gebru N.T., Guergues J., Verdina L.A. (2024). Fkbp5 gene deletion: circadian rhythm profile and brain proteomics in aged mice. Aging Cell.

[38] Rein T. (2016). FK506 binding protein 51 integrates pathways of adaptation: FKBP51 shapes the reactivity to environmental change. Bioessays.

[39] Nold V., Portenhauser M., Del Prete (2022). Impact of *Fkbp5* × early life adversity × sex in humanised mice on multidimensional stress responses and circadian rhythmicity. Mol Psychiatry.

[40] Wright K.P., Drake A.L., Frey D.J. (2015). Influence of sleep deprivation and circadian misalignment on cortisol, inflammatory markers, and cytokine balance. Brain Behav Immun.

[41] Choshen-Hillel S., Ishqer A., Mahameed F. (2021). Acute and chronic sleep deprivation in residents: cognition and stress biomarkers. Med Educ.

[42] Morgan E., Schumm L.P., McClintock M., Waite L., Lauderdale D.S. (2017). Sleep characteristics and daytime cortisol levels in older adults. Sleep.

[43] Brand S., Furlano R., Sidler M., Schulz J., Holsboer-Trachsler E. (2014). Associations between Infants’ crying, sleep and cortisol secretion and mother’s sleep and well-being. Neuropsychobiology.

[44] Ly J., McGrath J.J., Gouin J.P. (2015). Poor sleep as a pathophysiological pathway underlying the association between stressful experiences and the diurnal cortisol profile among children and adolescents. Psychoneuroendocrinology.

[45] Ota S.M., Kong X., Hut R., Suchecki D., Meerlo P. (2021). The impact of stress and stress hormones on endogenous clocks and circadian rhythms. Front Neuroendocrinol.

[46] Evans J., Silver R., Pfaff D.W., Volkow N.D., Rubenstein J. (2020). Neuroscience in the 21st Century: From Basic to Clinical.

[47] Bering T., Blancas-Velazquez A.S., Rath M.F. (2023). Circadian clock genes are regulated by rhythmic corticosterone at physiological levels in the rat hippocampus. Neuroendocrinology.

[48] Dib R., Gervais N.J., Mongrain V. (2021). A review of the current state of knowledge on sex differences in sleep and circadian phenotypes in rodents. Neurobiol Sleep Circadian Rhythms.

[49] Anderson S.T., Meng H., Brooks T.G. (2023). Sexual dimorphism in the response to chronic circadian misalignment on a high-fat diet. Sci Transl Med.

[50] Kon N., Yoshikawa T., Honma S. (2014). CaMKII is essential for the cellular clock and coupling between morning and evening behavioral rhythms. Genes Dev.

[51] Kon N., Sugiyama Y., Yoshitane H., Kameshita I., Fukada Y. (2015). Cell-based inhibitor screening identifies multiple protein kinases important for circadian clock oscillations. Commun Integr Biol.

[52] Silva A.J., Stevens C.F., Tonegawa S., Wang Y. (1992). Deficient hippocampal long-term potentiation in α-calcium-calmodulin kinase II mutant mice. Science.

[53] Bankhead P., Loughrey M.B., Fernández J.A. (2017). QuPath: open source software for digital pathology image analysis. Sci Rep.

[54] Williams T., Ruiz A.J., Ruiz A.M. (2022). Impact of APOE genotype on prion-type propagation of tauopathy. Acta Neuropathol Commun.

[55] Oh S.W., Harris J.A., Ng L. (2014). A mesoscale connectome of the mouse brain. Nature.

[56] So C.J., Miller K.E., Gehrman P.R. (2023). Sleep disturbances associated with posttraumatic stress disorder. Psychiatr Ann.

