# Supplementary material for: FKBP51 overexpression in the corticolimbic system stabilizes circadian rhythms
Source: Cell Stress Chaperones. 2024 Dec 12;30(1):22–32. doi: 10.1016/j.cstres.2024.12.003 (PMC11750455; doi:10.1016/j.cstres.2024.12.003)
Supplement: Supplementary file 1 — Supplementary material [file mmc1.docx]

**Supporting** **Information**

**FKBP51 Overexpression in the Corticolimbic System Stabilizes Circadian Rhythms**

Niat T. Gebru^1,2^, David Beaulieu-Abdelahad^1,2^, Danielle Gulick^1,2^, Laura J. Blair^1,2,3^

^1^Byrd Alzheimer's Center and Research Institute, Tampa, Florida, USA

^2^Department of Molecular Medicine, University of South Florida, Tampa, Florida, USA

^3^Research and Development, James A. Haley Veterans Hospital, Tampa, FL, USA

Correspondence:

Laura J. Blair

4001 E. Fletcher Ave.

Tampa, Florida 33613

(813) 396-0639

[laurablair@usf.edu](mailto:laurablair@usf.edu)

Danielle Gulick

4001 E. Fletcher Ave.

Tampa, Florida 33613

(813) 974-7402

[dgulick@usf.edu](mailto:dgulick@usf.edu)

**
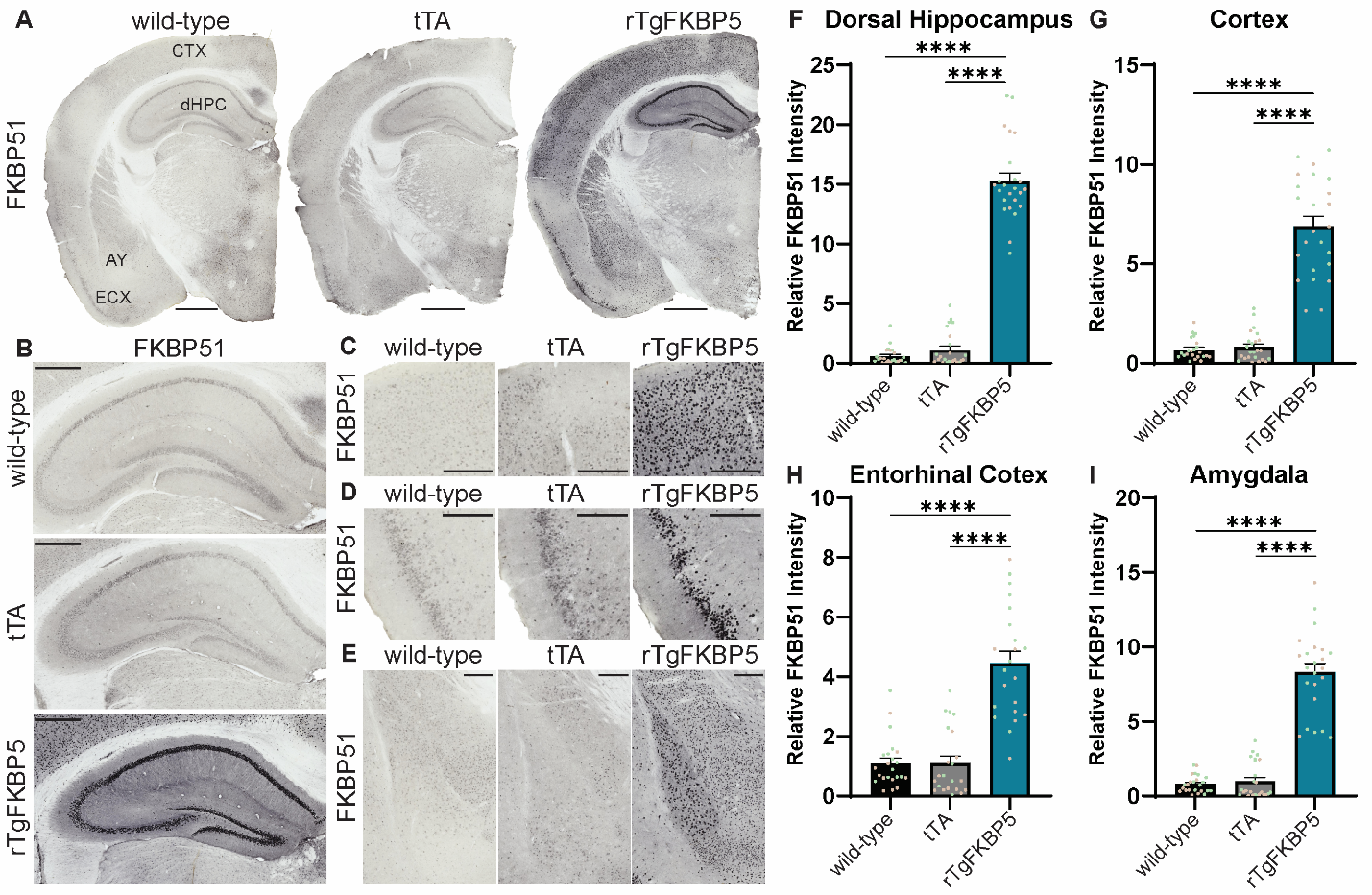
**

**Supplementary Figure 1: rTgFKBP5 mice express high levels of FKBP51 in the brain. (A)** Representative images of FKBP51 staining in hemibrains from wild-type, tTA, and rTgFKBP5 mice. Representative images and relative intensity quantitation of FKBP51 levels in the **(B, F)** hippocampus, **(C, G)** cortex (somatosensory), **(D, H)** entorhinal cortex, and **(E, I)** amygdala of wild-type, tTA and rTgFKBP5 mice. (n = 22–26/genotype) Males are represented in green and females in orange. Scale bar represents 800 µm for A, 400 µm for B, and 200 µm for C-E. Data were analyzed by one-way ANOVA with Tukey post hoc test. Statistical significance is indicated by ****p <0.0001.


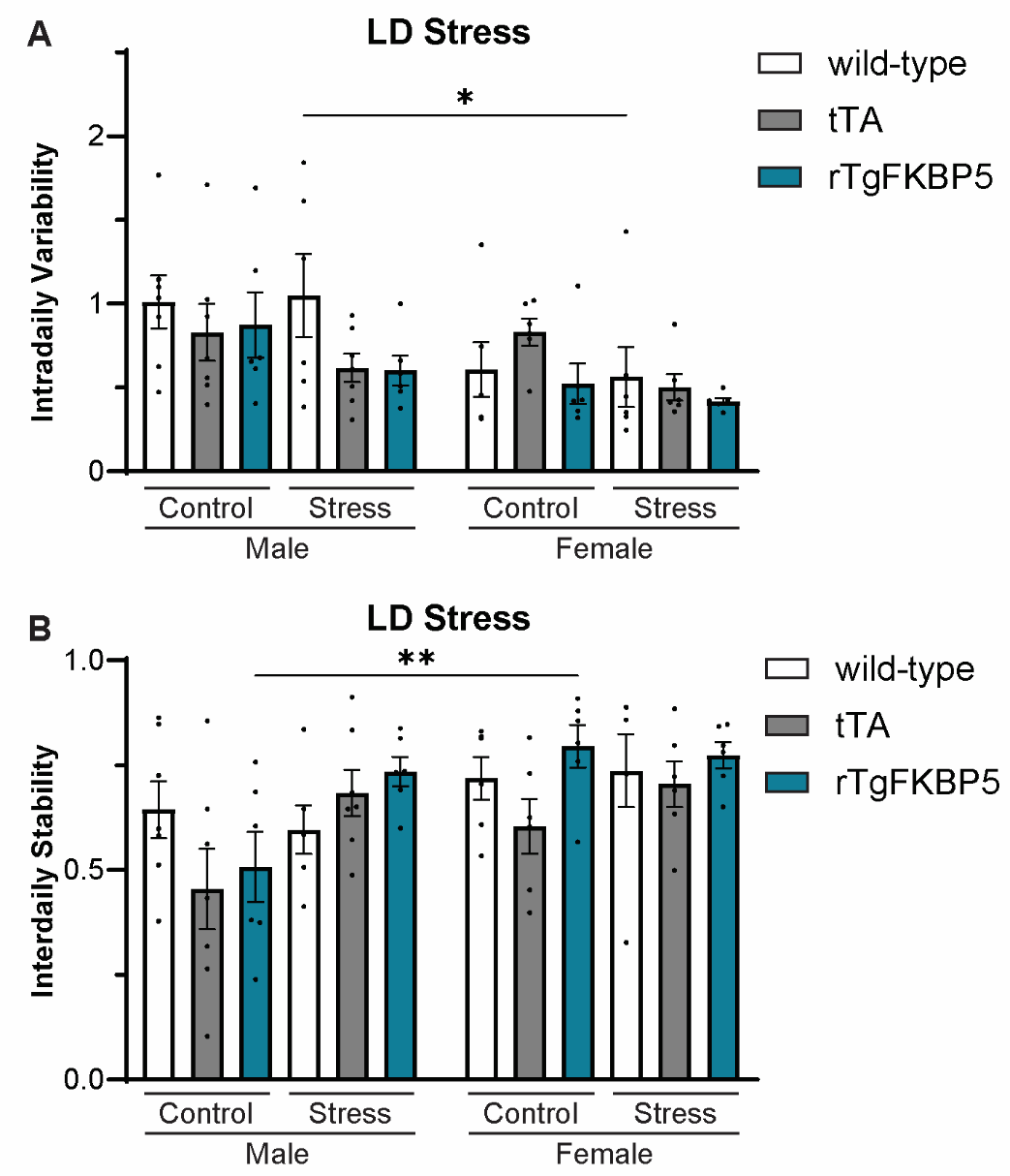


**Supplementary Figure 2: FKBP5 overexpression does not impact rhythm variability or stability.** Wheel-running activity was used to determine **(A)** Intradaily variability and **(B)** Interdaily stability in male and female wild-type, tTA, and rTgFKBP5 mice during LD stress. Data analyzed using SPSS MANOVA and ANOVA with Tukey post hoc test. Results represented as mean ± SEM (n =10-12/sex/genotype).
